# Supplementary material for: Substrate mediated nitridation of niobium into superconducting Nb2N thin films for phase slip study
Source: Sci Rep. 2019 Jun 19;9:8811. doi: 10.1038/s41598-019-45338-1 (PMC6584497; doi:10.1038/s41598-019-45338-1)
Supplement: Supplementary file 1 — Substrate mediated nitridation of niobium into superconducting Nb2N thin films for phase slip study [file 41598_2019_45338_MOESM1_ESM.pdf]

Supporting Material

Substrate mediated nitridation of niobium into superconducting Nb_2N thin films for phase slip study

Bikash Gajar[1,2], Sachin Yadav[1,2], Deepika Sawle[1,2], Kamlesh K. Maurya[1,3], Anurag Gupta[1,2], R.P. Aloysius[1,2], and Sangeeta Sahoo[*,1,2]*

[1] *Academy of Scientific and Innovative Research (AcSIR), AcSIR Headquarters CSIR-HRDC Campus, Ghaziabad, Uttar Pradesh 201002, India.*

[2]*Electrical & Electronics Metrology Division, National Physical Laboratory, Council of Scientific and Industrial Research, Dr. K. S Krishnan Road, New Delhi-110012, India.*

[3]*Indian Reference Materials Division, National Physical Laboratory, Council of Scientific and Industrial Research, Dr. K. S Krishnan Road, New Delhi-110012, India.*

**The correspondence should be addressed to S.S. (Email: sahoos@nplindia.org)*

Contents:

1. **AFM morphology for nitride samples B1, B2 and B3 and the Nb control samples B1^{Ox}, B2^{Ox} and B3^{Ox}.**
2. ***IVCs* for oxide samples B1^{Ox}, B3^{Ox} for both up and down current sweep directions.**
3. ***IVCs* for both up and down current sweep directions for nitride samples B1 and B2.**
4. ***R(T)* measurements in presence of perpendicular magnetic field for nitride samples B1, B2 and B3.**
5. **Calculation of Ginzburg-Landau (GL) coherence length (ξ_{GL}) for B1, B2 and B3.**
6. **Resistive tailing in the SC-state**
7. **Summary Table: Characteristic parameters for the nitride samples.**

1. AFM morphology for nitride samples B1, B2 and B3 and the Nb control samples $B1^{Ox}$, $B2^{Ox}$ and $B3^{Ox}$:

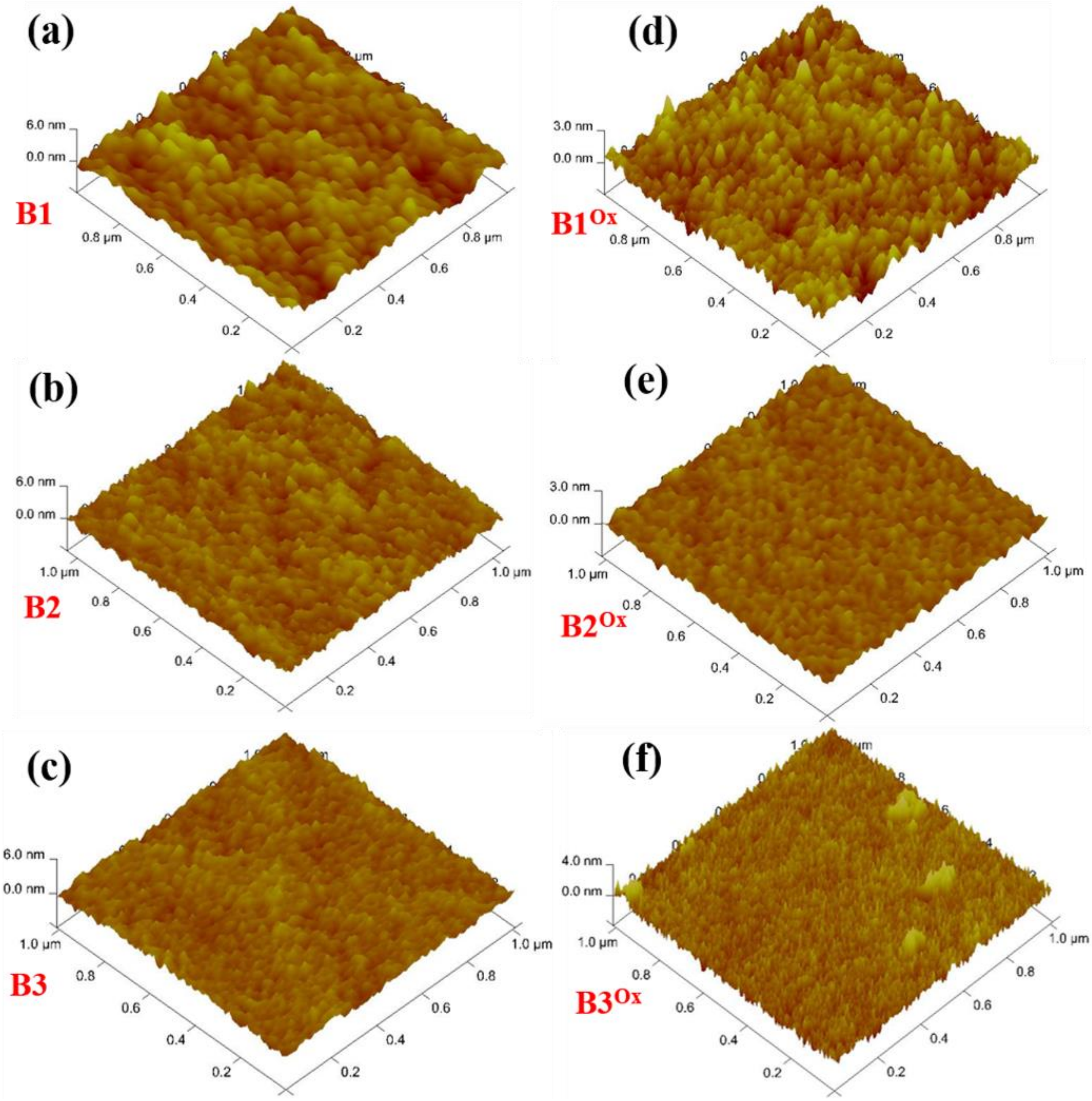

Fig. S1: *Morphological characterization. AFM topography images showing the granular nature of the films with variations in grain sizes for nitride samples B1, B2 and B3 in (a), (b) and (c), respectively and for the Nb control samples* $B1^{Ox}$*,* $B2^{Ox}$ *and* $B3^{Ox}$ *in (d), (e) and (f), respectively.*

AFM topography images, presented in Fig. S1, show the surface morphology of the nitride samples B1, B2 and B3 and the Nb control samples $B1^{Ox}$, $B2^{Ox}$ and $B3^{Ox}$. Both the nitride samples and the Nb control samples look granular in nature. The average grain sizes are estimated to be 40 nm, 30 nm and 23 nm for the nitride samples B1, B2 and B3, respectively. The overall variation in the grain size for the region bounded by the voltage probes are about ± 10 nm for all the three samples. Therefore, grain size reduces for reduced thickness. Further, the surface looks less uniform for the thinner samples than that appeared for the thicker one. On the other hand, the estimated average grain sizes are 37 nm, 27 nm and 12 nm for the control samples $B1^{Ox}$, $B2^{Ox}$ and $B3^{Ox}$, respectively with an overall variations of ± 7 nm for all of them. Grain sizes follow the same detrimental trend for the control samples also with reduction in thickness. For control samples, the reduction in T_c can be understood by the grain size reduction and for the thinnest sample $B3^{Ox}$ the grain size is much lower if we compare the same for the thinnest nitride sample B1. However, we do not observe staircase like phase slip lines (*PSLs*) in the current voltage characteristics (*IVCs*) for $B3^{Ox}$ and the *R(T)* was observed to be sharp with no such resistive tailing which was present for all the nitride samples.

2. IVCs for oxide samples B1^{Ox}, B3^{Ox} for both up and down current sweep directions:

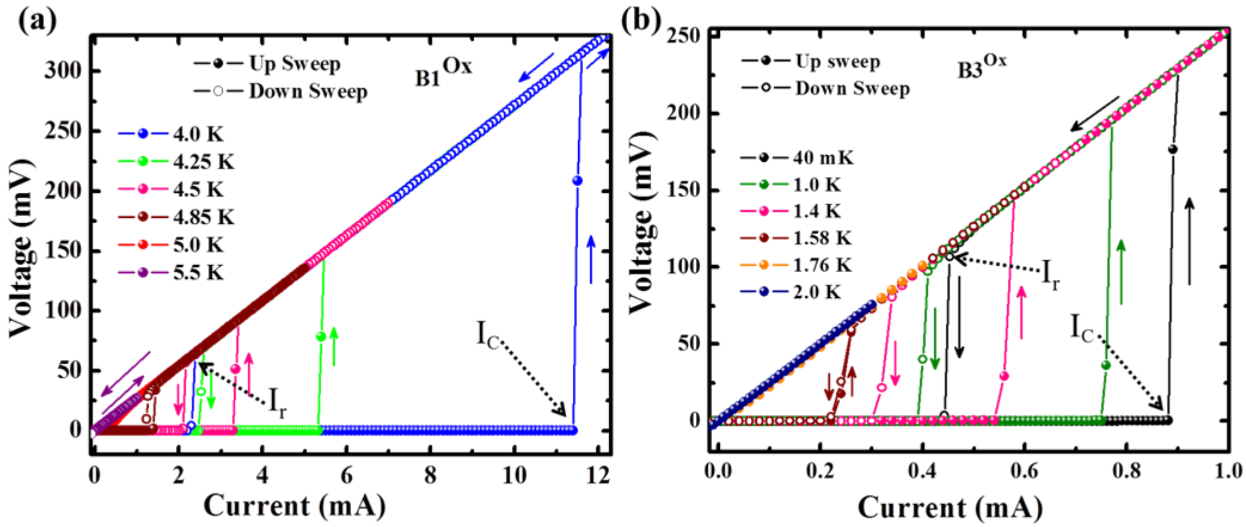

Fig. S2: Current voltage characteristics (IVCs) for oxide samples. *IVC isotherms for both up and down current sweep directions for B1^{Ox} (a) and B3^{Ox} (b). The critical current (I_C) and the retrapping current (I_r) are marked by the dotted arrows while the solid arrows show the sweep direction for the bias current.*

In Fig. S2, we have shown the *IVCs* for the oxide samples for both up and down sweep directions for the bias current. Here we selected the thickest (B1^{Ox}) and the thinnest (B1^{Ox}) samples among the three samples in order to observe the effects of thinning on the *IVCs* for the oxide samples. As we have already seen in the main text that for the nitride samples thickness plays a vital role in monitoring the transition region for superconductor-metal transition in their *R(T)* and also in *IVCs*. However, for the oxide samples we observe a single step sharp transition from superconductor to normal metallic transition at the critical current I_C. The same is observed in the reverse direction at the retrapping current Ir which is much less than I_C. Hence, the *IVCs* are hysteretic with respect to the current sweeping direction. Except for the values of these two characteristic currents I_C and I_r both the samples show similar type of hysteretic *IVCs* which

show a direct transition from SC to NM and *vice-versa*. It should be noted here that contrary to the nitride sample, we do not observe any intermediate resistive states for the oxide samples.

3. *IVCs* for both up and down current sweep directions for nitride samples B1 and B2:

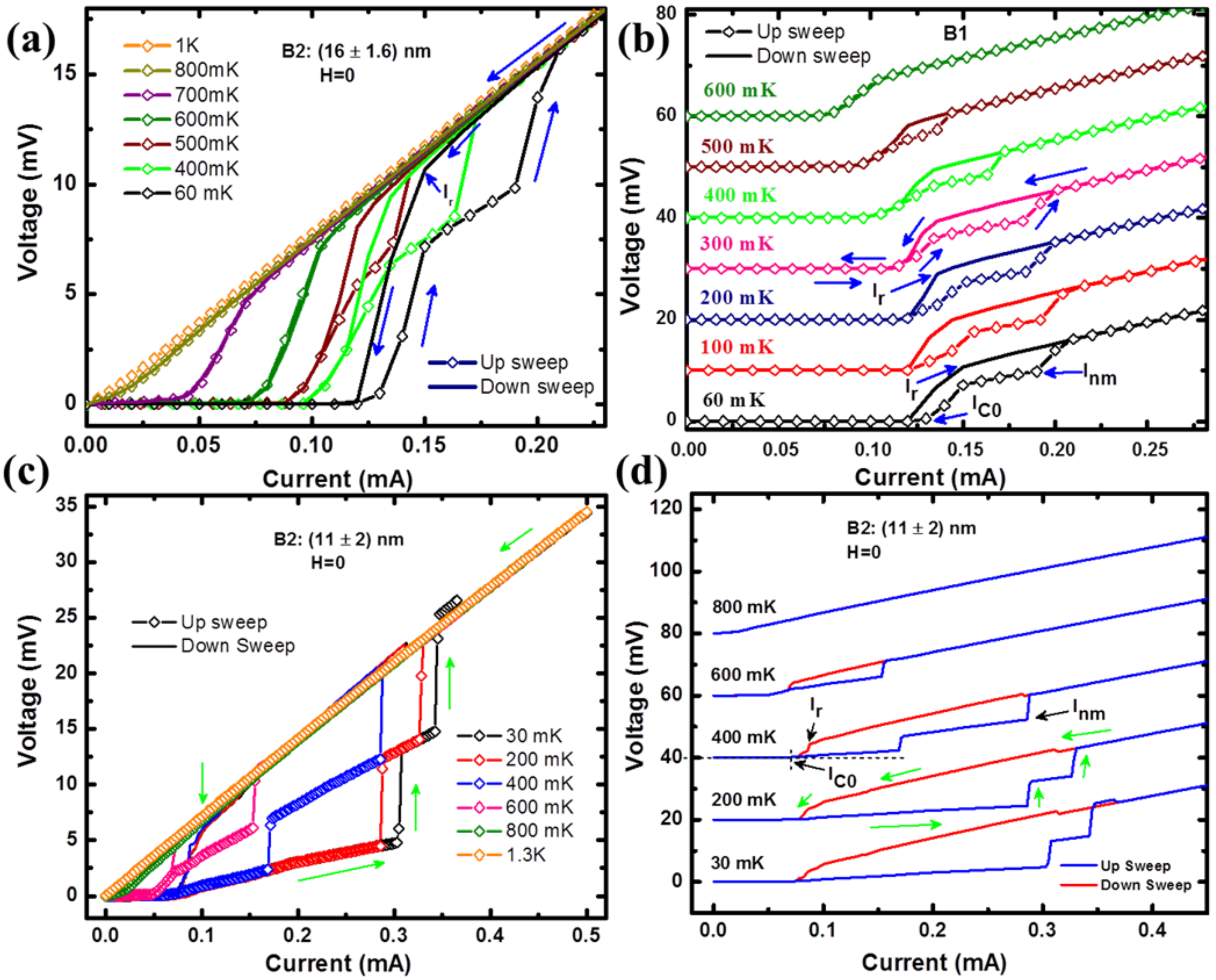

Fig. S3: Zero-field IVCs for nitride samples B1 and B2 for both increasing & decreasing current sweeping directions. *Isothermal IVCs for B1 (a) and for B2 (c). For clarity, the IVC isotherms are shifted in the voltage axis by 10 mV from the consecutive IVC isotherm for B1 (b) and by 20 mV for B2 (d), respectively. The superconducting state in each IVC corresponds to zero-voltage in the voltage axes presented in (b) & (d). The characteristic currents, namely, the retrapping current* I_r*, the critical currents* I_{c0} *&* I_{nm} *are defined by the arrows in (b) and (d).*

4. *R(T)* measurements in presence of perpendicular magnetic field for nitride samples B1, B2 and B3:

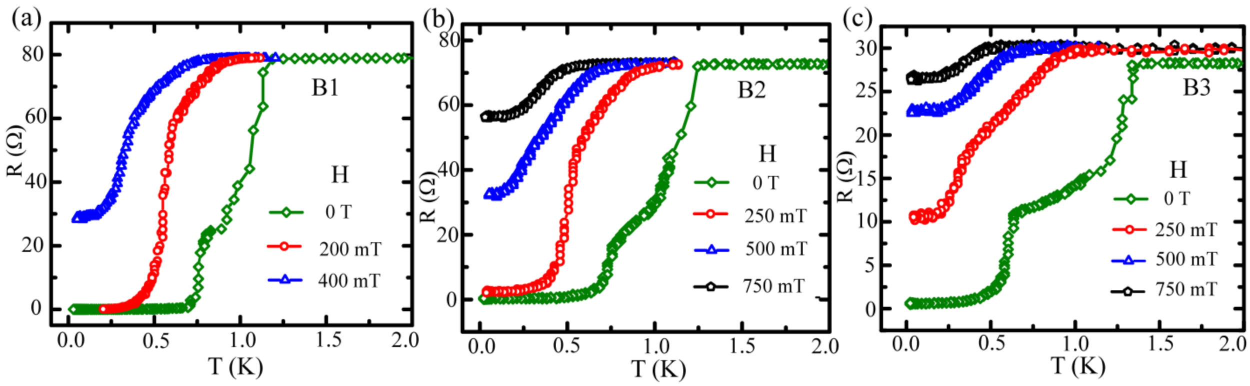

Fig. S4: Effects of perpendicular magnetic field on R(T) characteristics for nitride samples. *Field-dependent R(T) for B1 (a), B2 (b), and B3 (c), respectively.*

In the main text, we have discussed about zero-field *R(T)* measurements in detail for the nitride samples. In Fig. S4, we present field-dependent *R(T)* including the zero-field *R(T)* separately for all the three samples. Here the variations in the zero-field *R(T)* characteristics among the samples are clearly visible. The curvature in the region-I is getting much more prominent with reduction in thickness from samples from B1-to-B2 and from B2-to-B3. With application of an external magnetic field of about 200-250 mT, the curvature in region one gets almost reversed and the transition shifts towards lower temperature. The kink, separating the two regions as explained in detail in the main text, almost disappears with the application of field and the *R(T)* characteristics look smooth compared to that measured at zero—field. The curvature in region-II remains almost unchanged for all the samples when measured under the external field.

5. Calculation of Ginzburg-Landau (GL) coherence length (ξ_{GL}) for B1, B2 and B3:

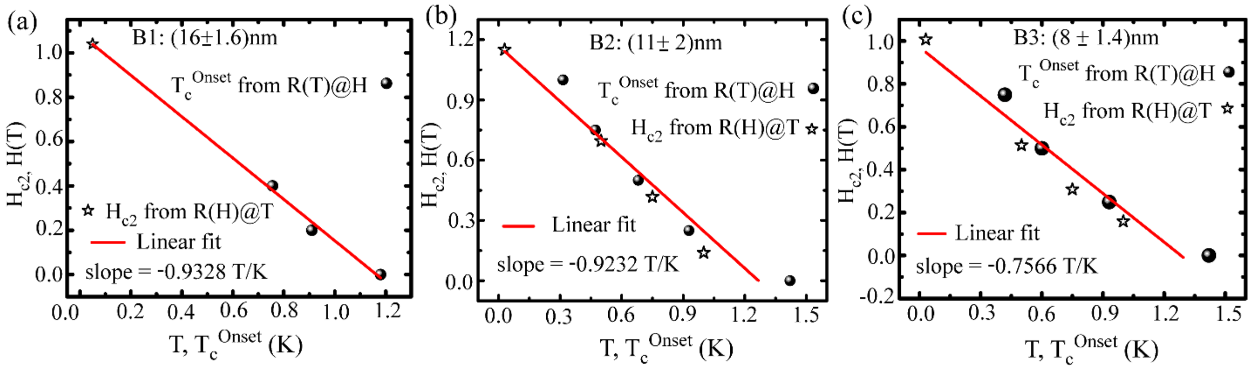

Fig. S5: H-T phase diagram for nitride samples. *Experimental points are obtained from magnetoresistance [R(H)] isotherms and from field dependent R(T)measurements under fixed external magnetic field applied perpendicular to the sample. The linear fit confirms that the samples are in dirty limit. The slope provides the GL coherence length* ξ_{GL}*, the details are explained in the text.*

We have calculated the Ginzburg-Landau coherence length, $\xi_{GL}(0)$, from the upper critical field $H_{c2}(Tesla)$ using the following formula[1,2], $\xi_{GL}(0) = \left[\frac{\phi_0}{2\pi T_c\left|\frac{dH_{c2}}{dT}\right|_{T_c}}\right]^{1/2}$, where ϕ_0 is the flux quantum. In order to estimate $\xi_{GL}(0)$, we have plotted the temperature dependent H_{c2} for all the three nitride samples in Fig. S5. We have obtained the values for H_{c2} from isothermal magnetoresistance *R(H)* measurements and the related points are marked as the star-shaped scattering points. Further, we have calculated T_c^{Onset} from the field dependent *R(T)* measurements

carried out under a fixed external magnetic field and the points are represented by the solid spherical scattering points in Fig. S5.The experimental data from both *R(H)* and *R(T)* measurements follow a linear variation between the critical field and the critical temperatures. The slope of the fit is used to calculate the GL coherence length, $\xi_{GL}(0)$. Here, we used $T_c^{Onset\text{-}I}$ as the T_c and accordingly, we have evaluated $\xi_{GL}(0)$ as 17.3 nm, 16.96 nm and 17.96 nm for B1, B2 and B3, respectively. As our thickness values for the nitride samples are of the order or less than the related coherence lengths, therefore, the samples can be considered in 2D limit.

6. Resistive tailing in the SC-state

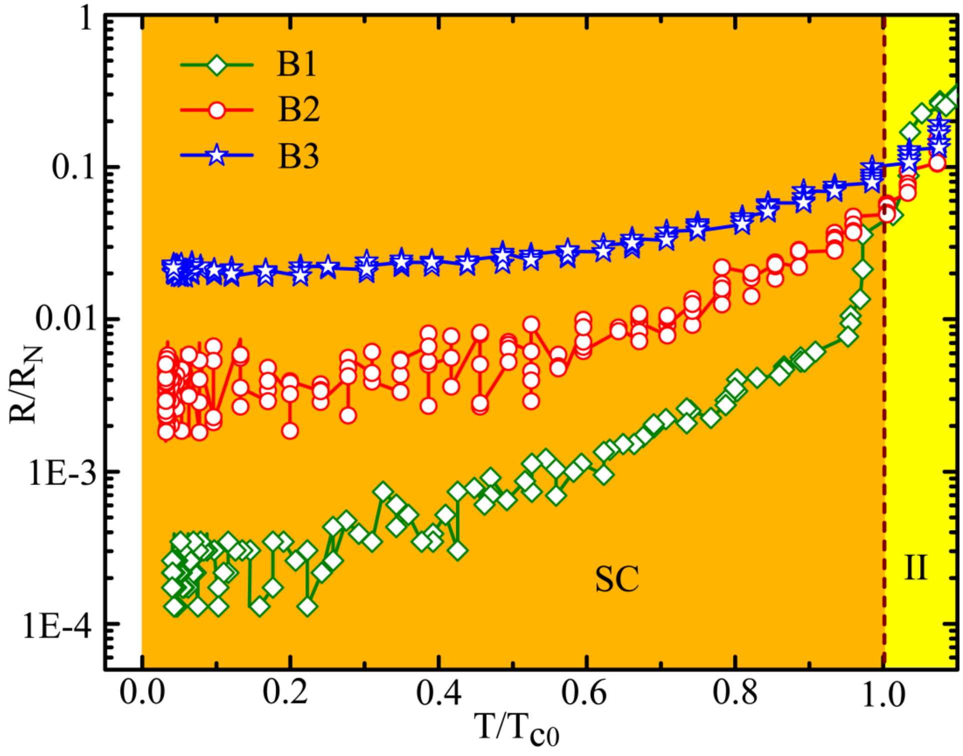

Fig. S6: *A collective representation of the SC-states in a semi-logarithmic plot to have the insight into the SC-state.*

7. Summary Table: Characteristic parameters for the nitride samples

Table 1: *Characteristic parameters for the nitride samples*

Sample	Thick-ness (nm)	Grain size (nm) ±10 nm	R_N (Ω)	$T_c^{Onset-I}$ (K)	$T_c^{Onset-II}$ (K)	T_{c0} (K)	T_C^{IV} (K)	ΔT_I (K)	ΔT_{II} (K)	ΔT_{II} /T_{C0}	R@ $T_c^{Onset-II}$ (Ω)
**B1**	16	40	79	1.18	0.82	0.73	0.8	0.36	0.09	0.123	**0.4 R_N**
**B2**	11	30	72.3	1.24	0.77	0.68	0.9	0.47	0.09	0.132	**0.25R_N**
**B3**	8	23	28.3	1.35	0.64	0.54	0.9	0.71	0.1	0.185	**0.3 R_N**

We have summarized the characteristic parameters that are obtained from the *R(T)* and *IVC* measurements for the nitride samples. The thickness and the grain sizes are measured by atomic force microscopy (AFM). Here, T_C^{IV} relates the transition temperature obtained from *IVC*s and it is mentioned in the main text as T_C. ΔT_I and ΔT_{II} correspond to the transition width in region-I and region-II respectively. It is clear that transition width is much wider in region-I than that in region-II. The normal state resistance R_N and the onset critical temperature $T_c^{Onset-I}$ vary in an unconventional way with the thickness. Further, the T_C^{IV} appears to be close to $T_c^{Onset-II}$ and from the *IVC*s, we have observed that below T_C^{IV} PSLs appear for all the samples. This indicates that the phase fluctuations in region-II and SC state contribute significantly to the resistive transition.

References:

1. Orlando, T. P., McNiff, E. J., Foner, S. & Beasley, M. R. Critical fields, Pauli paramagnetic limiting, and material parameters of Nb_3Sn and V_3Si. *Phy. Rev. B* **19**, 4545 (1979).
2. Bawa, A., Gupta, A., Singh, S., Awana, V. P. S., Sahoo, S. Ultrasensitive interplay between ferromagnetism and superconductivity in NbGd composite thin films. *Sci. Rep.* **6,** 18689 (2016).
